# Effects of the Daily Consumption of Stevia on Glucose Homeostasis, Body Weight, and Energy Intake: A Randomised Open-Label 12-Week Trial in Healthy Adults

**DOI:** 10.3390/nu12103049

**Published:** 2020-10-06

**Authors:** Nikoleta S. Stamataki, Benjamin Crooks, Abubaker Ahmed, John T. McLaughlin

**Affiliations:** 1 Division of Diabetes, Endocrinology & Gastroenterology, School of Medical Sciences, Faculty of Biology, Medicine and Health, Manchester Academic Health Sciences Centre, The University of Manchester, Oxford Rd, Manchester M13 9PL, UK; nikoleta.stamataki@manchester.ac.uk (N.S.S.); benjamin.crooks@postgrad.manchester.ac.uk (B.C.); Abubaker.Ahmed@mft.nhs.uk (A.A.); 2 Department of Gastroenterology, Salford Royal NHS Foundation Trust, Stott Lane, Salford M6 8HD, UK; 3 Department of Gastroenterology, Manchester Foundation Trust, Oxford Road, Manchester M13 9WL, UK

**Keywords:** stevia, non-nutritive sweeteners, glycaemia, body weight, energy intake

## Abstract

Stevia is a non-nutritive sweetener, providing sweet taste with no calories. This randomised, controlled, open-label 2-parallel arm trial examined the effects of daily stevia consumption on glycaemia in healthy adults. Secondary endpoints included body weight (BW) and energy intake (EI). Healthy participants (*n* = 28; aged 25 ± 5y, body mass index 21.2 ± 1.7 kg/m^2^) were randomised into either the stevia group (*n* = 14)—required to consume a stevia extract daily—or to the control group (*n* = 14). At weeks 0 and 12, the glucose and insulin responses to an oral glucose tolerance test were measured; BW and EI were assessed at weeks 0, 6, and 12. There was no significant difference in the glucose or insulin responses. There was a significant main effect of group on BW change (F(1,26) = 5.56, *p* = 0.026), as the stevia group maintained their weight as opposed to the control group (mean weight change at week 12: −0.22 kg, 95%CI [−0.96, 0.51] stevia group, +0.89 kg, 95%CI [0.16, 1.63] control group). The energy intake was significantly decreased between week 0 and 12 in the stevia group (*p* = 0.003), however no change was found in the control group (*p* = 0.973). Although not placebo-controlled, these results suggest that daily stevia consumption does not affect glycaemia in healthy individuals, but could aid in weight maintenance and the moderation of EI.

## 1. Introduction

There is a general consensus that overconsumption of caloric sugars, mainly through the consumption of sugar-sweetened beverages, leads to a greater energy intake and a poor diet quality, further associated with weight gain and/or type 2 diabetes mellitus (T2D) [1,2,3]. Non-nutritive sweeteners (NNS) represent a broad class of sweet compounds that are used in a variety of beverages and food products, providing a sweet taste yet contributing little or no energy to the diet. However, significant controversy exists regarding the effects of NNS consumption on body weight and metabolic health outcomes [4,5,6,7], with effects ranging from harmful to neutral to beneficial. The discrepancy between study outcomes has been attributed to methodological limitations [8], while significant issues as to how the evidence base on NNS is generated, interpreted, and communicated by the expert community also exist [9]. The need for more long-term randomised trials on the effects of NNS consumption on metabolic health outcomes and body weight is emerging.

A number of studies have explored the potential of NNS use to influence acute metabolic responses and especially the blood glucose response. The results of those studies have been systematically reviewed, overall showing a neutral effect of NNS on glucose control [10,11,12]. One hypothesis tested was based on the premise that the human body associates sensory cues with metabolic responses, so the activation of sweet taste receptors in the oral and extra-oral tissues might alter glucose metabolism through promoting insulin and/or incretin release. Although experimental data using human cell lines and animal models consistently show sweet taste receptor activation leading to increased insulin and incretin release in vitro [13,14], the results from human trials have not confirmed this [15]. One factor may be the very high doses of NNS used in non-human studies. In addition, following in vitro demonstrations that treatment with NNS could enhance glucose uptake via the upregulation of transporters [16], it was hypothesised that the concomitant consumption of NNS and carbohydrates would result in higher glucose response, but again human trials have failed to show such an acute effect [17,18]. Overall, NNS consumed as single agents or concomitantly with carbohydrates do not seem to affect acute glucose response, apart from a few reports showing small effects on either the glucose response [18] or glucagon-like peptide-1 (GLP-1) secretion [19]. However, it remains unclear as to whether repeated exposure to NNS would have any effects on glucose homeostasis in the long term.

Stevia (steviol glycosides) has gained great popularity as a natural NNS alternative to caloric sugars, nevertheless it remains the least studied in terms of its effects on human metabolic responses. There is some evidence suggesting that stevia might assist with glucose regulation. Gregersen et al. showed that the concomitant consumption of stevioside with a full meal reduced the postprandial incremental area under the curve (iAUC) of blood glucose compared to control (maize starch) in individuals with T2D [20]. In healthy adults, a reduction in postprandial iAUC for glycaemia and insulinaemia was also demonstrated when a stevia-sweetened beverage was consumed along with a meal, compared to consuming a sugar-sweetened beverage [21]. The long-term consumption of rebaudioside A (one type of steviol glycoside) did not alter the fasting blood glucose in subjects with T2D [22] or with glucose intolerance [23]. To the best of our knowledge, currently there is no available study investigating whether there is a change in glucose response to an oral glucose tolerance test (OGTT) after the daily consumption of stevia in healthy adults.

Despite providing minimal energy, NNS have paradoxically been suggested to be involved in weight gain and T2D risk in cohort studies. However, meta-analyses of randomised controlled trials (RCTs) indicate that body weight is slightly but significantly reduced with NNS use [6,7]. In line with these conclusions, we have previously shown a beneficial effect of consuming a stevia-sweetened beverage prior to lunch on short-term appetite and total energy intake [24]; whether this effect is sustained with prolonged use is yet to be examined. Only a few RCTs have investigated the long-term effects of the consumption of stevia on body weight so far, with all showing no significant change [25,26,27].

The aim of this study was to investigate the effects of the daily consumption of stevia for 3 months, taken in doses similar to real-life consumption, on the glucose homeostasis, body weight, and energy intake in healthy adults with a normal body mass index (BMI). The primary outcome was change in postprandial glucose response before and after the intervention, while secondary outcomes included change in body weight and energy intake.

## 2. Materials and Methods

### 2.1. Study Design

A randomised, controlled, open-label 2-parallel-arm trial was conducted. Participant assignment was based on a random sequence generated via an online tool (www.random.com) by an independent researcher and was pre-stratified by gender to ensure a balance between the two arms of the trial. The research protocol was reviewed and approved by the University of Manchester Research Ethics Committee (2018-4812-7661); all subjects signed informed consent prior to participation and were compensated for their time at the end of the trial. The trial is registered at clinicaltrials.gov under the registration NCT03993418.

### 2.2. Participants

Healthy adults with a normal BMI (18.5–25 kg/m^2^), aged 18–40 years old, who were non-habitual consumers of NNS (≤1 can of diet beverages per week or ≤1 sachet of NNS per week) and non-restrained eaters (restraint eating score in the Dutch Eating Behaviour Questionnaire (DEBQ) ≤ 3) were recruited. Other inclusion criteria were fasting blood glucose ≤ 6 mmol/L, stable weight for the last 12 months (±5 kg), willingness to comply with the study protocol, no self-reported food allergy or intolerance to foods supplied during the study. Exclusion criteria were being on a diet or having ceased a diet in <4 weeks, following any special diets for weight maintenance, being vegetarian or vegan, alcohol consumption more than 14 units a week, more than 10 h of vigorous physical activities per week and/or planning to increase or decrease physical activity levels in the future, having ceased smoking in the last 6 months, and female participants who are or may be pregnant or currently lactating.

The participants who were interested in participating contacted the researchers via email and were then sent a link to an online screening questionnaire. Eligible participants from the online screening questionnaire were invited to a screening session that was scheduled on a morning after an overnight fast. During this session, fasting blood glucose, weight, and height measurements were conducted to ensure that the participants met the inclusion criteria for the study. In addition, the participants needed to agree to be allocated to either treatment group. Participants who were found to be eligible and agreed to participate were consented then randomised into one of the study groups.

Sample size calculation was conducted for the primary outcome, glucose response to an OGTT measured by iAUC, and was based on data of a previous trial in healthy subjects that involved the ingestion of glucose load (mean iAUC 117 mmol/L × 120 min, SD: 41 mmol/L × 120 min) [28]. With 28 subjects, there was an 80% power to detect a 20% change in the iAUC, which is considered a clinically significant change in glucose response for the current study design, assuming a within-person correlation of 0.5, α of 0.05 and taking into consideration the study design (2 groups × 2 measurements).

In total, 68 participants completed the online survey and 36 attended the screening session. Thirty-one participants were randomised to the 2 study groups, and all study procedures took place between January 2019 and December 2019. A detailed flowchart can be found in the Supplementary Material Figure S1. Withdrawals were due to time constraints or significant changes in the participants’ daily routine and not because of known study-related adverse effects.

### 2.3. Protocol

The participants in the stevia group were given a commercially available stevia drops product (SweetLeaf Stevia Sweet Drops Clear, SweetLeaf^®^, Wisdom Natural Brands, Arizona, USA) and were instructed to consume 5 drops twice daily with their habitual drinks (5 drops of stevia corresponds to the sweetness of one teaspoon of table sugar). The choice of stevia drops over any other powder product was taken based on the purity of the drops, containing only stevia leaf extract in water, as in most commercially available powder product stevia is usually mixed with a bulking agent (erythritol, inulin etc.), and therefore any effects shown would not be indicative solely of stevia. The participants were advised to use the stevia drops with their coffee, tea, smoothie, porridge, juice, or other beverage according to their preferences, ideally before lunch and before dinner. Advice on sugar consumption was not given. The participants allocated to the control group were not required to change anything in their usual diet. Both groups were advised to keep their physical activity levels consistent for the duration of the study and to avoid consuming any products containing NNS.

**Figure 1:**
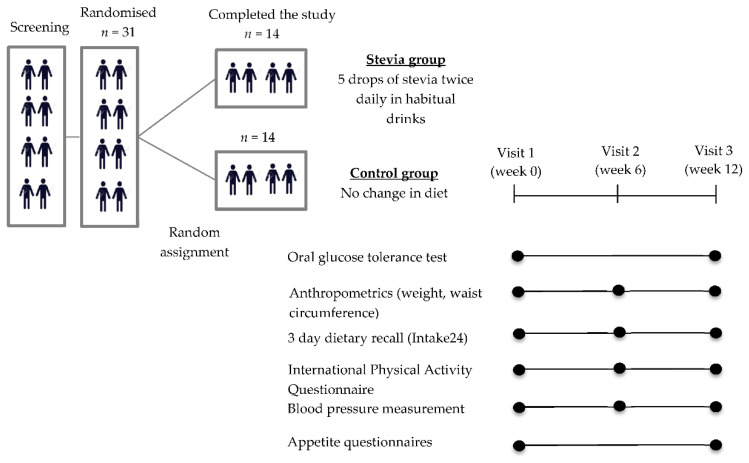
Description of the study design and outcomes.

The participants were required to attend 3 study sessions—visit week 0 (baseline), visit week 6, and visit week 12. All the study procedures were conducted at the Neuroscience and Psychiatry Unit, University of Manchester. A graphical description of the study design and the schedule of the assessments can be found in Figure 1.

Visit week 0 and visit week 12 were conducted on the morning after an overnight fast. The participants were required to refrain from any vigorous physical activities and alcohol consumption the day before testing and to consume their evening meal before 22:00 the night before each visit. Upon arrival, a cannula was inserted into a forearm vein for repeated blood sample collection and a baseline blood sample was collected. Next, the participants ingested 75 g of glucose dissolved in 250 mL of tap water. The glucose beverages were prepared on the morning of testing by the researchers, and were served in transparent beakers as colourless liquids at room temperature. Blood samples were then collected 15, 30, 45, 60, 90, and 120 min after the consumption of the glucose load.

Immediately following the collection of a blood sample, the glucose levels were determined using a HemoCue Glucose 201+ Analyzer (HemoCue, Angelholm, Sweden). A HemoCue cuvette was placed into a droplet of whole blood, then the cuvette was wiped clean and placed in the cuvette holder to be measured.

The remaining blood samples were placed into serum-separating vacutainers. The tubes for serum separation were allowed to clot at room temperature for 30 min before centrifuging for 15 min at 3000 rpm and 4 °C. After centrifuging, the serum samples were aliquoted into labelled Eppendorf tubes at −80 °C until analysis.

For visit week 6, the participants did not have to fast, but it was scheduled at least 2 h away from a main meal (i.e., at least 2 h after breakfast or after lunch). The assessments conducted at visit week 6 can be found in Figure 1.

Body weight was determined by a digital scale (SECA 813 electronic scale with a large platform) in light clothes without shoes, and height was measured using a portable stadiometer (SECA 213 Portable Height Measure, Hamburg, Germany). Waist circumference was measured via a measuring tape (SECA 201 Ergonomic Circumference Measuring Tape, Hamburg, Germany). Blood pressure was measured twice and the average value was recorded (OMRON M2 Basic). The energy and macronutrient intake were assessed via 24 h diet recalls. The participants were required to complete 3 diet recalls (2 weekdays and 1 day on a weekend) before each study visit (before visit week 0, week 6, and week 12). The diet recalls were performed using a free open-source self-completed computerised dietary recall system, Intake24 (https://intake24.co.uk/). The validity of Intake24 against interviewer-led 24 h recalls has been established (the mean intakes of all macronutrients and micronutrients were within 4% of the interviewer-led recall) [29]. All the participants received a training session with a dietician on how to recall their food intake using this system. Physical activity was monitored via the International Physical Activity Questionnaire (IPAQ—long lasting 7-day self-administered format).

The participants filled out a three-factor eating questionnaire (TFEQ), measuring their perceptions of dietary restraint, disinhibition, and hunger [30], and a control of eating questionnaire (CoEQ) (only the subscales of sweet craving, savoury craving) at visit week 0 and at visit week 12 [31].

### 2.4. Analyte Assays

Serum insulin was determined by a sandwich ELISA method using a commercially available human insulin kit (ab20001—Insulin Human SimpleStep Elisa Kit, Abcam, Cambridge, UK) with a minimum sensitivity of 1.9 pmol/L. The insulin concentration was measured at the 0, 30, 45, 60, and 120 min time points. All of the samples for a particular participant (pre and post intervention) were measured on the same ELISA plate.

### 2.5. Compliance

The participants allocated to the stevia group were given a diary to fill out every day that included information on whether they had their two doses of stevia, what they had it with, and their reason for not taking it if they skipped a dose. A compliance percentage was calculated for each participant using the information provided from the diary. We considered it adequate adherence when >80% of the prescribed stevia was consumed. The participants were also required to bring the stevia bottle with them at visit week 6 and at visit week 12. The weight of the bottle was measured and compared to a reference one (the drops that should have been used by week 6 and week 12 were counted and removed from the reference bottle). 

### 2.6. Statistical Analysis

Descriptive statistics were used for variables such as age, weight, height, BMI, and questionnaire and are presented as means ± SDs. The rest of the data are presented as means ± SEs. Mixed-model repeated measures ANOVAs were used to assess the glucose and insulin responses, body weight, anthropometrics, dietary intake, physical activity levels, and questionnaire scores. Session (week 0 and week 12) and time (0–120 min) were within-subject independent variables and study group (stevia or control) was the between-group independent variable. Where significant main effects or interactions were found, we followed up with post hoc comparisons using Bonferroni-corrected criteria. In cases where the sphericity was violated, the Greenhouse–Geisser-corrected *p* values are reported. Statistical significance was determined at *p* ≤ 0.05. Analyses were conducted by using IBM SPSS Statistics Version 25.

## 3. Results

### 3.1. Baseline Characteristics

There were no differences in the baseline characteristics between groups. The baseline characteristics of the participants who completed the trial are presented in Table 1. There was no difference between the treatment groups in terms of age, BMI, weight, waist circumference, or eating behaviour traits assessed via the DEBQ (restraint, emotional and external eating). The estimated compliance based on the participants’ diaries was 95 ± 5%. All the participants’ bottles weighed very close to the reference bottle (±3 g) both at week 6 and at week 12.

### 3.2. Glucose Response

**Figure 2:**
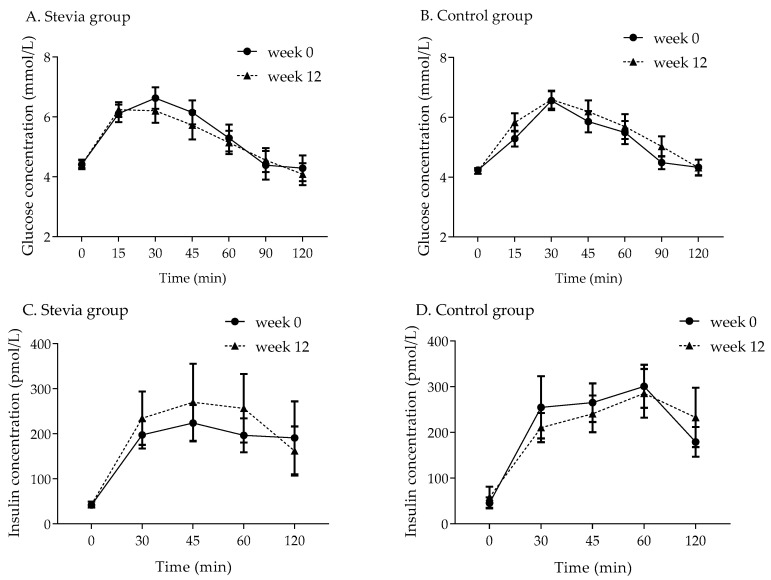
Blood glucose and serum insulin concentrations during the oral glucose tolerance tests for participants in the stevia group (*n* = 14, panels A and C) and in the control group (*n* = 14, panels B and D) at baseline (week 0) and after 12 weeks of intervention. Values are means ± SEs. Venous blood samples could not be collected from one participant in the stevia group, and the serum insulin was not measured for this participant, *n* = 13 for 2C.

The week 0 and week 12 blood glucose values from the OGTTs are presented in Figure 2 (panels A and B). All the participants had fasting blood glucose within the normal range (3.9–5.5 mmol/L). There were no significant differences in glucose response between the two groups, and no main effect of treatment group or interaction between the session (week 0 and week 12) and treatment group. The iAUC for glycaemia in the stevia group was (mean ± SE) 132 ± 31.2 mmol/L × min at baseline and 133 ± 29.5 mmol/L × min at week 12; in the control group, these values were 131 ± 19.1 mmol/L × min and 159 ± 33.1 mmol/L × min, respectively. The peak glucose concentration at baseline in the stevia group was 7.08 ± 0.34 mmol/L and at week 12 it was 6.91 ± 0.32 mmol/L; in the control group, these values were 6.66 ± 0.31 mmol/L and 6.82 ± 0.29 mmol/L, respectively.

### 3.3. Insulin Response

There were also no significant differences in insulin response among the study groups, and no significant main effect of the treatment group or interaction between the treatment group and session (week 0 and week 12). The week 0 and week 12 serum insulin values are presented in Figure 2 (panels C and D). The iAUC for insulin response to the OGTT was 16.5 ± 3.59 nmol/L × min at baseline and increased to 19.3 ± 5.88 nmol/L × min at week 12; however, this increase was not statistically significant (*p* = 0.516, paired samples *t*-test), as it was driven by two single subjects. Similarly, there was no difference in iAUC for insulin response in the control group at baseline and after 12 weeks, and the values were 21.6 ± 3.55 and 20.4 ± 4.07 nmol/L × min, respectively. The peak insulin concentration was 336 ± 78.9 pmol/L at baseline in the stevia group and 347 ± 95.9 pmol/L at week 12; in the control group, these values were 362 ± 62.8 and 367 ± 63.5 pmol/L, respectively.

### 3.4. Body Weight and Other Anthropometric Indices

**Figure 3:**
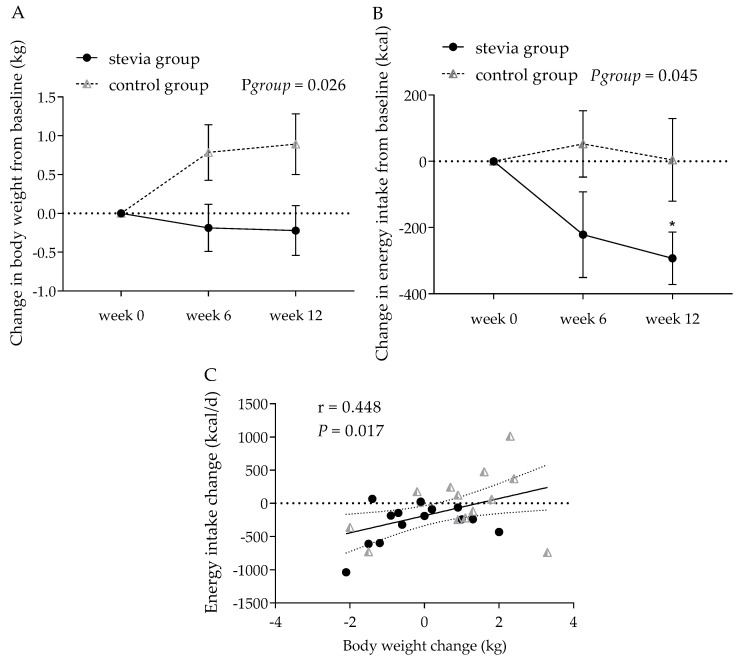
Change in body weight (**A**) and energy intake (**B**) in the stevia and control groups over 12 weeks (*n* = 14 in each group). Differences in body weight were correlated with changes in energy intake (**C**). Data are expressed as means ± SE. * *p* = 0.003.

The change in body weight was significantly different between the two groups (main effect of treatment group, F(1, 26) = 5.56, *p* = 0.026, η^2^p = 0.176; session-by-treatment group effect, F(2, 52) = 3.43, *p* = 0.040, η^2^p = 0.117) (Figure 3). There was a statistically significant increase in body weight over the 12-week trial for participants in the control group (mean weight change: 0.56 kg, 95% CI [0.13, 0.99] in the control group) compared to the participants in the stevia group (−0.14 kg, 95% CI [−0.56, 0.29]). Including the baseline weight as a covariate, the results were further strengthened (treatment group effect, F(1, 25) = 6.07, *p* = 0.021, η^2^p = 0.195; session-by-treatment group interaction, F(2, 50) = 3.68, *p* = 0.032, η^2^p = 0.128).

Table 2 shows the BMI, waist circumference, and blood pressure values for the stevia and control groups at week 0, week 6, and week 12 of the intervention. There was a significant main effect of session (week 0, 6, and 12) on the BMI change from baseline (F (1, 26) = 4.95, *p* = 0.035, η^2^p = 0.160), but no other significant changes were observed for waist circumference, systolic and diastolic blood pressure, or pulse rate.

### 3.5. Energy Intake

The self-reported energy and macronutrient intake data are shown in Table 3. The energy intake at week 0 was not significantly different between treatments (*p* = 0.929). The change in energy intake from week 0 is presented in Figure 3b, showing a significant main effect of the treatment group (F(1, 26) = 4.43, *p* = 0.045, η^2^p = 0.146), with participants in the stevia group reporting a significantly reduced energy intake over the 12-week trial period (mean energy intake change: −171 kcal, 95% CI [−303, −39.9]) compared to the control group (18.9 kcal, 95% CI [–112, 150]). Further exploration of the data revealed that, in the stevia group, energy intake was significantly lower at week 12 relative to week 0 (*p* = 0.003, Bonferroni correction for multiple comparisons, *p* ≤ 0.008), however no difference was found in the control group (*p* = 0.973). The individual differences in weight change between the treatment groups were significantly correlated with the individual differences in energy intake change at week 12 (*r* = 0.448, *p* = 0.017) (Figure 3c). The reduction in self-reported daily energy intake observed in the stevia group was not because of selectively reducing a specific macronutrient such as sugars or carbohydrates, but was an overall reduction in energy intake, since no differences were observed between the two groups in terms of their carbohydrate, fat, protein, sugar, or fibre intakes before and after the intervention (Table 3).

### 3.6. Physical Activity

There was no difference in physical activity levels assessed via the IPAQ between week 0, week 6, and week 12 in the two treatment groups. In the stevia group, the total MET min per week were calculated (mean ± SE) as 3346 ± 574.3 at week 0, 3256 ± 574.4 at week 6, and 3133 ± 352.3 at week 12. In the control group, the total MET min per week were 4415 ± 1047 at week 0, 3579 ± 754.9 at week 6, and 4373 ± 1393 at week 12. There were no main effects or interactions found (all *p* > 0.05).

### 3.7. Appetite Expression

The results from the TFEQ and CoEQ are presented in Table 4. The participants in the control group reported overall higher hunger scores on the TFEQ subscale compared to the stevia group (main effect of treatment group F(1, 26) = 4.64, *p* = 0.041, η^2^p = 0.152); no difference was found for the restraint and disinhibition subscales of the TFEQ between groups or between week 0 and week 12. The participants in the control group also reported an overall higher craving for savoury scores on the CoEQ subscale compared to the stevia group (main effect of treatment group F(1, 26) = 8.96, *p* = 0.006, η^2^p = 0.256); there was also a significant interaction between the session (week 0 and week 12) and the treatment group (F(1, 26) = 4.83, *p* = 0.037, η^2^p = 0.157), showing a reduction in self-reported craving for sweet in the control group. However, this difference did not reach statistical significance after correcting for multiple comparisons (*p* = 0.040, Bonferroni correction for multiple comparisons *p* ≤ 0.025). Exploratory correlation analysis revealed a significant positive correlation between the sweet craving ratings and sugar intake (g/1000 kcal) in the control group (*r* = 0.419, *p* = 0.027), but not in the stevia group (*r* = 0.225, *p* = 0.013).

## 4. Discussion

The present study investigated the effects of the daily consumption of stevia drops for 12 weeks on glucose response, body weight, and energy intake in healthy adults. We observed no difference in the glucose and insulin response, however the stevia and control groups showed distinct patterns in body weight and energy intake. The stevia-consuming participants did not significantly alter their body weight from baseline, but did not demonstrate the weight gain that occurred in the control group. Participants in the stevia group also reported a lower total energy intake during the trial compared to the controls, while the physical activity levels did not change across the intervention period.

The primary outcome of this trial was glucose response, assessed via OGTTs performed at baseline and after 12 weeks of intervention in healthy individuals without diabetes. No significant difference was observed with regard to the treatment group or intervention time. These findings support our understanding of the effects of NNS in general on glycaemia. When NNS are consumed alone, no difference in glucose levels has been reported so far in acute single-exposure trials in humans [32,33,34,35,36]. This observation probably shows that the activation of sweet taste receptors by NNS does not exert any clinically relevant effects on glucose homeostasis signalling in the context of human consumption. In addition, no significant change in glucose response has been observed in acute studies where NNS were consumed along with a glucose load by healthy non-obese adults [17,18,19]. The rationale behind this was based on data from in vitro demonstrations showing that treatment with NNS might enhance glucose uptake due to the upregulation of the glucose transporters. Therefore, an increase in glucose response would be anticipated when carbohydrates were consumed concomitantly with NNS compared to being consumed alone. This hypothesis was not confirmed by human studies in healthy participants [17]. However, each NNS is a distinct chemical compound and has its own biological fate in the human body, which might influence individual NNS responses [37]. Effects on biological targets other than sweet taste receptors cannot be discounted. Regarding stevia, two studies have provided evidence that it might assist with glucose regulation, as lower postprandial levels of glucose were observed following the consumption of a meal supplemented with stevia in healthy adults [21] or in patients with T2D [20]. These results are further supported by the demonstration of enhanced pancreatic beta-cell function by steviol glycosides [38]. However, no significant difference was observed in the glucose and insulin response when stevia was ingested alone [24,39]. The direct administration of rebaudiana A (type of steviol glycoside) in the duodenum of healthy adults also did not result in incretin release [40]. A recent meta-analysis of RCTs investigating the effects of long-term stevia consumption on metabolic markers showed no significant difference in fasting blood glucose in favour of steviol glycosides; the doses of consumption varied between 3.75 mg/kg/day and 1500 mg/day of stevioside [41]. No significant change has been demonstrated for fasting insulin following long-term stevia consumption [22,42]. In the present study and in line with the majority of results from human trials, the daily consumption of commercially available stevia did not influence glucose homeostasis or insulin response in healthy adults.

In line with our results, similar effects have been demonstrated by long-term RCTs in healthy adults using other types of NNS. No effect on glucose, insulin, and GLP-1 responses was observed in the study by Higgins et al. [43] following 12 weeks of aspartame consumption in two different doses (350 mg and 1050 mg/d) compared to a placebo. Furthermore, no change in glucose and insulin response was found in the study by Grotz et al. [44], which investigated the effects of 12 weeks of sucralose consumption (1000 mg/day) against a placebo group in normoglycaemic males or following 7 days of sucralose administration (780 mg/d) in healthy subjects [45]. On the other hand, lower insulin sensitivity has been demonstrated in two studies following daily sucralose consumption [46,47], and a recent study by Dalenberg et al. [34] also showed that consuming 7 sucralose-sweetened beverages not without carbohydrates over 10 days decreased insulin sensitivity in healthy human volunteers. Whether this is a sucralose-specific effect needs further investigation. In the present study, there was no difference in the insulin response to an OGTT before and after 12 weeks of daily stevia consumption compared to the control group in healthy adults.

One a priori secondary outcome was change in body weight, assessed at week 6 and week 12 of the intervention period. In this trial, we demonstrated that the participants allocated to the stevia group maintained their body weight compared to the control group, who showed a significant increase in body weight, which could be attributed to a general trend towards weight gain by the population. Further, the results from the self-reported energy intake, which was another secondary outcome, showed a decrease in energy intake at week 12 of the intervention in the stevia group, but not in the control group. Even though the change in body weight does not match the change in energy intake, since a reduction in energy intake should indicate weight loss, there was a significant correlation between individual changes in body weight and individual changes in energy intake. Participants were not placed on an energy-restricting diet, and physical activity levels were kept stable throughout the trial; the only guidance provided for those in the stevia group was that they should consume the stevia drops daily, ideally in a drink or a hot beverage before lunch and before dinner. With this advice, we attempted to reproduce the design of a previous acute study on stevia effects on food intake, where a significant reduction in total energy intake was demonstrated when consuming a stevia-sweetened preload prior to lunch compared with consuming water or caloric beverages [24]. If that effect would be sustained and not compensated for in the next meals, that could explain the lower energy intake results, as observed in the present study. However, opposed to our results, another recent study evaluated the effects of daily rebaudiana A consumption for 12 weeks against another three types of NNS and sucrose on body weight and energy intake and showed no effect on both measures [25]. The lack of a difference in body weight following encapsulated stevioside consumption by people with mild hypertension and patients with T2D was also shown by another two studies [22,27]. If the effects of stevia on energy intake and body weight are mediated by sweetness per se, no effect would be expected in trials where oral sweet taste is bypassed. More randomised long-term trials powered with body weight and energy intake as primary outcomes are required to confirm these initial findings of stevia consumption effects in healthy adults, but also in populations for whom weight loss and reduction in energy intake is crucial—i.e., individuals with overweight, obesity, T2D, or metabolic syndrome.

Recent research now focuses on elucidating the effects of NNS consumption on brain systems related with appetite and reward. The ingestion of glucose induces decreased activity in the hypothalamus, a change typically linked to satiety signalling by the brain [48]. However, sweet taste in the absence of nutritive carbohydrates does not seem to elicit a similar response in the hypothalamus [49,50]. Differences between nutritive sweeteners and NNS have been also demonstrated during taste activation, with both stimuli showing the activation of the primary gustatory cortex, anterior insula, and frontal operculum. However, during NNS tasting the reward centres remained unresponsive [51,52]. The above results indicate that NNS might not have similar satiating effects in the brain as nutritive sweeteners. On the other hand, differences in the neural processing of sweet taste in the brain among regular NNS consumers have also been reported, showing a potential adaptation in brain systems following repeated exposure to NNS. In particular, regular consumers of diet soda have shown greater activity patterns in reward regions of the brain during the consumption of nutritive and nonnutritive sweet tastes, compared to non-diet soda consumers [53]. We should highlight again the potential of different types of NNS exhibiting differential responses, yet further studies are needed to explore brain responses following stevia consumption in humans.

The findings of this research also raise the question of whether there was any behavioural change between the two groups that could have led to the observed distinct effects on body weight and energy intake. The participants in the control group reported higher hunger scores on the TFEQ, independent of intervention time. Hunger is the conscious experience associated with the drive to eat. Even though perceived hunger might not predict intake, it has been shown to predict an individual’s ability to manage their body weight or the success of a weight-loss program [54]. Higher hunger scores were associated with greater body size in another study [55]. However, this difference in susceptibility to hunger ratings was a baseline difference between the two groups, was not influenced by the intervention period, and was a self-reported questionnaire measure; thus, any observations remain exploratory at this point. Interestingly, we observed a positive correlation between sweet craving ratings and sugar intake in the control group; a reduction in sugars intake was associated with a reduction in the subjective feeling of sweet craving. This was a spontaneous unexpected change in eating behaviour in the control group, who were not following any dietary guidance. However in the stevia group the sugar intake stayed relatively stable and so did the sweet cravings, in line with previous results showing a protective effect of NNS beverages against craving-induced increases in energy intake [56]. Further research is required to explore eating behavioyr changes induced by the introduction of NNS into the diet of habitual and non-habitual NNS consumers.

The strengths of this study include the real-life scenario design, and participants were allowed to make choices and adjust the addition of the stevia drops to their daily routine. The dose chosen was also realistic, and could simulate the regular consumption of stevia by the general population. Among the limitations of this study is the fact that the results are only specific to the conscious consumption of stevia at this point. The increase in body weight in the control group should be interpreted with caution, since other factors such as menstrual cycle or hydrating status might be responsible for this outcome, however these potential confounding factors were the same for the two groups and the gender ratio was the same. Another limitation of the study could be the use of a natural history control group (no treatment) instead of a null control group receiving placebo drops. It has been documented that participants randomised to placebo-control conditions in obesity research studies often report improved outcomes that are similar to those of people receiving the active treatment, even when the individual is aware that they are receiving a placebo [57,58]. A control for sweetness could be another NNS arm, such as saccharin, aspartame, sucralose, etc., but that was out of the scope of this trial at this time. It is likely that sweet taste may mediate these results, suggesting that a double-blind design delivering the sweetener bypassing the oral cavity could be compared to an open-label design (sweet taste perception included) to investigate this hypothesis. This study was powered to detect a significant difference in the primary outcome, glucose response, not secondary outcomes where the two groups showed distinct effects, and therefore a powered for body weight and/or energy intake randomised controlled trial should be pursued, especially in populations where the reduction in energy intake is critical, such as in individuals with overweight and/or obesity, metabolic syndrome, or T2D.

In summary, our data provide evidence that the daily consumption of stevia in real-life doses does not affect glycaemia in healthy normal-weight individuals, but could aid in weight maintenance and the moderation of energy intake. More research is warranted to explore these promising findings further.

## Figures and Tables

**Table 1:** Baseline characteristics.

	Stevia Group (*n* = 14)	Control Group (*n* = 14)	*p* Value
Age, y	25 (6)	25 (4)	0.795
Weight, kg	59.50 (9.00)	57.83 (7.98)	0.428
Height, m	1.65 (0.09)	1.67 (0.08)	0.934
BMI, kg/m^2^	21.71 (1.81)	20.73 (1.46)	0.122
Waist circumference, cm	71.64 (6.53)	70.57 (5.81)	0.651
Female (count)	11	11	
BMR (kcal) ^1^	1379 (197)	1368 (181)	0.880
Daily energy needs (kcal) ^2^	1930 (276)	1915 (253)	0.882
DEBQ scores			
Restraint eating	2.06 (0.43)	1.90 (0.59)	0.484
Emotional eating	2.26 (0.63)	2.28 (0.45)	0.947
External eating	3.09 (0.56)	3.11 (0.43)	0.930

Values are mean ± SD. BMR, Basal Metabolic Rate; DEBQ, Dutch Eating Behaviour Questionnaire. ^1^ Calculated using the Mifflin–St Jeor equation, ^2^ calculated using BMR and a physical activity factor of 1.4.

**Table 2:** Anthropometric measures for the stevia and control groups over the 12-week intervention.

	Stevia Group (*n* = 14)			Control Group (*n* = 14)			*p* Values		
	Week 0	Week 6	Week 12	Week 0	Week 6	Week 12	Session	Group	Session × Group
Body weight, kg	59.50 (2.40)	59.31 (2.40)	59.27 (2.49)	57.83 (2.13)	58.61 (2.32)	58.72 (2.23)	0.289	0.769	0.040
Δ Body weight, kg	-	−0.19 (0.30)	−0.22 (0.32)	-	0.79 (0.36)	0.89 (0.39)	0.289	0.026	0.040
BMI, kg/m^2^	21.71 (0.48)	21.64 (0.48)	21.62 (0.49)	20.73 (0.39)	20.99 (0.42)	21.03 (0.40)	0.389	0.247	0.053
Δ BMI, kg/m^2^	-	−0.07 (0.11)	−0.09 (0.12)	-	0.26 (0.13)	0.31 (0.14)	0.388	0.035	0.054
Waist circumference, cm	71.64 (1.75)	71.93 (1.61)	71.11 (1.65)	70.57 (1.55)	71.18 (1.66)	71.00 (1.66)	0.135	0.783	0.199
Systolic blood pressure, mmHg	119.14 (2.46)	117.71 (3.60)	118.07 (2.78)	114.00 (1.95)	114.21 (2.81)	112.71 (2.58)	0.732	0.191	0.792
Diastolic blood pressure, mmHg	67.57 (1.76)	64.57 (1.51)	65.93 (1.17)	70.11 (1.24)	68.79 (1.51)	69.29 (1.93)	0.159	0.069	0.750
Pulses, beats per min	72.39 (2.52)	69.57 (2.46)	74.75 (2.14)	70.07 (1.49)	74.14 (3.66)	73.68 (2.33)	0.264	0.889	0.165

Values are mean ± SE. Δ calculated as change from week 0. BMI, body mass index.

**Table 3:** Energy and macronutrient intake levels in the stevia and control groups during the intervention.

	Stevia Group (*n* = 14)			Control Group (*n* = 14)			*p* Values		
	Week 0	Week 6	Week 12	Week 0	Week 6	Week 12	Session	Group	Session × Group
Energy intake, kcal × d^−1^	1659 (102.9)	1437 (106.2)	1366 (115.6)	1674 (137.7)	1727 (154.4)	1678 (167.2)	0.185	0.224	0.114
Δ Energy intake, kcal × d^−1^		−221.2 (129.3)	−292.8 (78.81)	-	52.52 (100.3)	4.36 (124.8)	0.185	0.045	0.114
Carbohydrates, g	193 (10.9)	175 (16.6)	170 (12.3)	205 (15.2)	206 (16.2)	208 (23.3)	0.643	0.164	0.533
Carbohydrates, %	47.9 (2.52)	48.7 (2.61)	51.6 (2.81)	50.3 (2.48)	48.7 (1.86)	49.4 (2.47)	0.586	0.975	0.440
Fats, g	67.6 (7.12)	53.8 (5.00)	52.3 (6.66)	66.4 (7.55)	68.7 (8.77)	66.00 (10.0)	0.241	0.338	0.174
Fats, %	35.9 (1.91)	33.4 (1.83)	32.8 (1.96)	34.8 (1.82)	34.8 (1.80)	34.1 (2.05)	0.500	0.787	0.691
Proteins, g	71.3 (8.99)	69.3 (7.11)	61.3 (7.00)	68.1 (6.82)	71.8 (6.82)	73.8 (4.92)	0.739	0.644	0.224
Proteins, %	16.8 (1.53)	19.9 (2.39)	17.9 (1.40)	16.5 (1.14)	16.8 (1.00)	18.9 (1.61)	0.282	0.635	0.253
Sugars, g/1000 kcal	49.3 (3.61)	49.8 (3.59)	49.2 (3.71)	49.7 (6.24)	43.5 (5.16)	38.1 (5.03)	0.395	0.230	0.435
Fibres, g/1000 kcal	8.29 (0.79)	8.49 (1.11)	7.79 (0.64)	7.54 (1.15)	8.53 (1.20)	8.10 (0.87)	0.487	0.911	0.648

Values are mean ± SE. Δ calculated as change from week 0.

**Table 4:** Appetite expression questionnaires for the stevia and control groups during the intervention.

	Stevia Group (*n* = 14)		Control Group (*n* = 14)		*p* Values		
	Week 0	Week 12	Week 0	Week 12	Session	Group	Session × Group
*TFEQ subscales*							
Cognitive restraint	5.93 (0.84)	5.86 (0.64)	4.79 (1.23)	6.36 (1.40)	0.226	0.819	0.186
Disinhibition	4.36 (0.74)	4.79 (0.70)	5.21 (0.59)	5.57 (0.54)	0.192	0.354	0.904
Hunger	4.86 (0.82)	4.86 (0.76)	7.36 (0.74)	6.79 (0.82)	0.504	0.041	0.504
*CoEQ subscales*							
Cravings for savoury	32.3 (3.67)	36.2 (4.50)	52.6 (5.33)	49.9 (4.27)	0.845	0.006	0.243
Cravings for sweet	39.3 (4.96)	40.4 (4.72)	46.9 (5.16)	36.1 (5.19)	0.087	0.807	0.037

Values are mean ± SE. TFEQ, three-factor eating questionnaire; CoEQ, control of eating questionnaire.

## Supplementary Materials

The following are available online at https://www.mdpi.com/2072-6643/12/10/3049/s1: Figure S1: participant flow chart.
