# Supplementary figures and images for: Effects of the Daily Consumption of Stevia on Glucose Homeostasis, Body Weight, and Energy Intake: A Randomised Open-Label 12-Week Trial in Healthy Adults
Source: Nutrients. 2020 Oct 6;12(10):3049. doi: 10.3390/nu12103049 (PMC7600789; doi:10.3390/nu12103049)

## Supplemental Material

Figure S1. Participant flow chart.

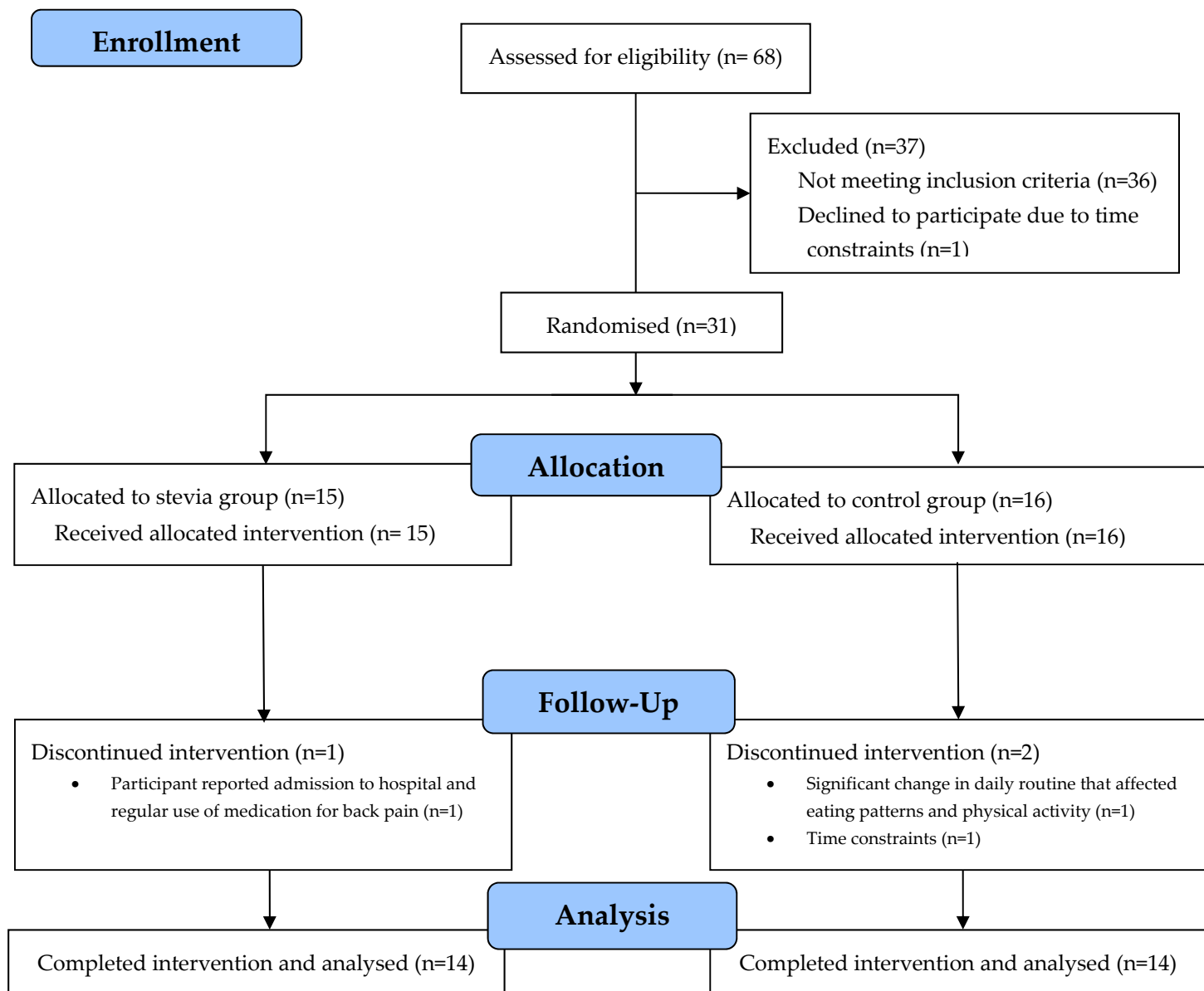

Supplement: Supplementary file 1 [file nutrients-12-03049-s001.pdf]
